# Andrographolide Alleviates Liver Damage Caused by *Salmonella* in Mice by Inhibiting the PANoptosis Pathway

**DOI:** 10.3390/microorganisms14040936

**Published:** 2026-04-21

**Authors:** Quanying Li, Limin Hou, Luna Su, Xiaoyu Wang, Yifan Zhu, Binghu Fang

**Affiliations:** College of Veterinary Medicine, South China Agricultural University, Guangzhou 510642, China; 19195356562@163.com (Q.L.);

**Keywords:** andrographolide, *Salmonella*, liver injury, PANoptosis

## Abstract

The emergence of antibiotic resistance in pathogens, including *Salmonella typhimurium*, poses a major challenge to animal health and safety. Andrographolide is well known for its antibacterial properties and therefore offers potential as an antimicrobial treatment to lessen the damage caused by *Salmonella*. PANoptosis is defined as an inflammatory coordinated cell death pathway encompassing apoptosis, pyroptosis, and necroptosis. To reduce the organ and tissue damage caused by bacterial infection and reduce antibiotic resistance, this study investigated the effect of andrographolide on liver damage in *Salmonella*-infected mice. We used a mouse model infected with *Salmonella typhimurium* for in vivo experiments, which involved the detection of the bacterial load in the liver, liver injury indicators, and expression of related PANoptosis-related genes and proteins. Here, our finding indicated that andrographolide effectively inhibited markers associated with apoptosis, pyroptosis, and necroptosis in mouse hepatocytes, alleviated liver injury and clinical symptoms caused by *Salmonella typhimurium* in mice, and thus exerted therapeutic effects. In this study, we observed that andrographolide modulated the markers associated with these three pathways, indicating their involvement in PANoptosis. These results suggest that andrographolide significantly relieve *Salmonella*-induced liver injury by inhibiting PANoptosis, highlighting the potential significance of andrographolide as an effective drug for the treatment of *Salmonella*.

## 1. Introduction

*Salmonella typhimurium* is a Gram-negative species with more than 2600 different serotypes that pose a serious risk to both animals and humans. The serotypes can largely be classified as nontyphoidal or typhoidal *Salmonella.* Typhoidal *Salmonella* can cause systemic infections with an extremely high fatality rate, whereas infections caused by non-typhoid *Salmonella* are typically self-limiting [1]. Their common characteristic is that they are transmitted mainly through the fecal–oral route. The main clinical symptom is diarrhea after animals and humans consume contaminated food [2]. As a result, *Salmonella* infections are considered among the most common diseases worldwide, causing approximately 100 million infections and more than 200,000 deaths per year [3]. Antibiotic treatment for *Salmonella* infections is currently a very serious problem. During antibiotic treatment, antimicrobial resistance (AMR) is associated with the misuse of antimicrobial drugs [4], such as therapeutic or preventive drugs for animals, including veterinary medicines for the treatment of diseases and growth promoters for use in animal husbandry. Moreover, the consumption of contaminated food or drink also greatly negatively affects people’s health. Second, the overuse of antibiotics can cause the bacteria to develop resistance. Therefore, more attention should be paid to the risks associated with antimicrobial misuse. *Salmonella* infection in mice can cause systemic infection and damage various organs. The liver is among the most important immune organs and plays roles in detecting, capturing and removing pathogens [5]. When animals or humans are infected with *Salmonella* and left untreated, in addition to intestinal damage, a significant degree of liver damage can occur [6]. This usually leads to an inflammatory response. Currently, antibiotics are the treatment of choice for salmonellosis. However, owing to antibiotic resistance and antibiotic residues, alternative treatments need to be considered. Some studies have shown that the functions of the intestine and liver affect each other through the gut–liver axis [7]. For instance, *Salmonella* uses bacterial hair adhesion molecules to attach to the surface of intestinal epithelial cells and invade subcutaneous tissues to proliferate [8]. Based on these findings, we hypothesized that endotoxin released by intestinal-colonized *Salmonella* may cause hepatic injury through the portal vein.

PANoptosis has been defined as a novel form of programmed cell death (PCD) that is regulated by multifaceted PANoptosome complexes with key characteristics: mutual crosstalk among pyroptosis, apoptosis, and necroptosis [9]. These three PCD patterns were previously considered in isolation. However, recently, a highly interrelated form of PCD called PANoptosis has been defined and has been associated with microbial infections, cancer, and autoinflammatory diseases [10]. According to the conclusions of several previous studies, “pan-apoptotic” cases are almost always a complex collection of multiprotein complexes (PANoptosomes), which are important for initiating cell death and sensing PAMPs, DAMPs, or other risk factors [11]. Moreover, it is important to note that PANoptosis is not merely the simultaneous occurrence of these three cell death pathways; rather, it is driven by a multiprotein complex known as the PANoptosome (e.g., comprising ZBP1, RIPK3, and caspase-8), which integrates signals from these pathways into a unified cell death program. This concept distinguishes PANoptosis from the simultaneous but independent occurrence of these three cell death modalities, emphasizing instead their interdependent interplay within a shared molecular platform. The morphological features of apoptosis include cell shrinkage, nuclear condensation, DNA fragmentation, and plasma membrane blebbing [12]. Apoptosis is caused mainly by death receptors (external pathway) or by the involvement of mitochondria (internal pathway). Pyroptosis is triggered mainly by bacterial or pathogen infection and is characterized by cell swelling and plasma membrane lysis, resulting in the formation of CASP-dependent pores and the subsequent release of proinflammatory molecules. Necroptosis is characterized by early loss of plasma membrane integrity, swelling of mitochondria, and leakage of cytosolic constituents [13]. Recent studies have indicated that cell damage caused by *Salmonella* infection is associated with PANoptosis: for example, pyroptosis and apoptosis drive the expulsion of infected intestinal epithelial cells (IECs) to limit the replication of *S*. *typhimurium* [14]. In addition, PANoptosis plays roles in liquid metabolism, redox regulation, and other essential cellular functions. Based on the above, we can determine whether the liver is associated with the mechanism of infection in the gut, both of which involve PANoptosis.

Diseases caused by bacteria are becoming more difficult to treat with antibiotics; therefore, the use of natural plant monomers for the treatment of inflammation has become an interesting topic of conversation. Andrographolide, a labdane-diterpenoid lactone, was reported to be an active component of *A. paniculata*. There are 44 species within the genus *Andrographis*, among which *Andrographis paniculata* is a multi-branched, upright, dark green annual herb. The plant was originally used for pain relief and anti-inflammatory purposes in the tropical and subtropical regions of India and has since been widely adopted for use in China and other Southeast Asian countries [15]. It is well known that andrographolide has a good effect on the treatment of liver disease. Additionally, the antibacterial activity of andrographolide has been extensively documented in the literature. Dafur et al.’s studies confirmed its effectiveness against various bacterial strains, including *Escherichia coli*, *Klebsiella pneumoniae*, and *Staphylococcus aureus* [16]. Specific MIC values reported include 125 μg/mL against *Mycobacterium tuberculosis* H37Rv and 125–500 μg/mL against multidrug-resistant *M. tuberculosis* strains [17]. Regarding *Salmonella typhimurium*, while direct MIC values are not available, andrographolide has been shown to enhance *Salmonella*-specific antibody responses and induce cell-mediated immunity against salmonellosis in murine models [18]. Therefore, it possesses antiviral, antithrombotic, hepatoprotective, anticancer and anti-inflammatory properties. According to the literature, andrographolide has a complex hepatoprotective mechanism that acts on multiple proteins in a cell-dependent manner [19]. Therefore, andrographolide has favorable protective and preventive effects against liver-related diseases. However, there is a paucity of reports in this area, and further research is needed. Therefore, based on the above research findings, this study will use andrographolide for the treatment of *Salmonella*-infected mouse livers. However, the findings suggest that the toxicity of andrographolide is inextricably linked to its dosage and cumulative dose, and that the metabolism of the drug varies greatly from species to species. The effects of andrographolide on humans should be treated with caution [20]. Therefore, we reviewed the relevant literature and rigorously selected the appropriate dosage.

Currently, bacterial drug resistance is a growing concern; therefore, the use of natural plant monomers to treat bacterial infections has become an interesting topic. However, the available data on the effects of andrographolide on *Salmonella* have been derived mainly from studies using synthetic andrographolide, rhubarb extract and silver nanoparticles against *Salmonella* infections [21,22,23]. Therefore, more information on the effects of andrographolide on *Salmonella*-infected animals is lacking. *Salmonella* is a well-known intestinal colonizer. Here, we hypothesize that PANoptosis is involved in liver damage from *Salmonella* infection. We therefore conducted experiments with mice exposed to *Salmonella* to explore the single and combined effects on the liver and examined the mechanisms involved with a focus on PANoptosis. Overall, our findings demonstrate that andrographolide helps mitigate *Salmonella*-induced liver damage, which may provide a reference for the use of other drugs to alleviate *Salmonella*-induced liver damage. The term PANoptosis has been used to describe an inflammatory, coordinated cell death pathway that includes apoptosis, pyroptosis, and necroptosis. Our study demonstrates that andrographolide modulates these three pathways, suggesting that they may be involved in PANoptosis.

## 2. Materials and Methods

### 2.1. Animal Treatment

All animal experiments conducted as part of this study were approved by the Ethics Committee at South China Agricultural University. Andrographolide was procured from Shanghai McLean Biotechnology Co., Ltd. (Shanghai, China). Kunming mice (40 total) were purchased from Guangzhou Medical University Laboratory Animal Center (Guangzhou, China) and were randomly assigned into five groups (n = 8 per group) using a computer-generated random number table: the blank control group (without any treatment fed for one week), the *Salmonella*-infected group, 50 mg/kg andrographolide (Andro) group, 100 mg/kg (Andro) group, and the 150 mg/kg (Andro) group, which were subjected to force-feeding and gavage once a day. The sample size of 8 mice per group was chosen based on previous studies [24], which have shown that this sample size is sufficient to detect significant differences in histopathological and biochemical outcomes. In addition, no animals were excluded from the final analysis. All animals survived until the end of the experiment. The doses of andrographolide (50, 100, and 150 mg/kg) were selected based on previously published studies demonstrating efficacy in various disease models, including ischemic stroke [25] and cancer xenograft models, as well as dose–response considerations. All the mice were fed under identical conditions and administered andrographolide at varying concentrations for 4 days, and the first three days were administered 100 μL of *Salmonella* solution at a concentration of 1 × 10^9^ CFU by gavage. Weight measurements should be taken and recorded every 3 days, while liver specimens are collected and stored at −80 °C. All the samples were randomly selected for use in subsequent experiments.

### 2.2. Real-Time Quantitative PCR Assay (RT-qPCR)

Gene expression was detected using RT-qPCR, as described in a previous study [26]. Total RNA was extracted from liver tissue using TRIzol reagent, which was used to lyse the samples and release the RNA. Then, two-phase separation with chloroform was performed to separate the RNA from the sample and the RNA was precipitated with isopropyl alcohol, cleaned with 75% ethanol, and then dissolved in DEPC water. The obtained RNA was subsequently measured using a spectrophotometer (Thermo Fisher Scientific, Waltham, MA, USA), diluted and reverse transcribed into cDNA. Reverse transcription was performed under the following thermal cycling conditions: reverse transcription at 50 °C for 5 min, followed by enzyme inactivation at 85 °C for 5 s. The primers and probes were purchased from the Assay on Demand (FAM) product line by Applied Biosystems and were used for real-time quantitative PCR (Bio-Rad Laboratories, Hercules, CA, USA) in accordance with the manufacturer’s operating procedures. The Ct values of the target genes were corrected by the GAPDH value, and all the experiments involved at least 3 replicates. The data are presented as Ct values and used to obtain deltaCt (dCt) values, which are expressed as fold change ± standard error of the mean. The primers used are listed in Supplementary Table S1.

### 2.3. Western Blotting

As outlined in a previous study, proteins were extracted from the livers of mice, and Western blotting was subsequently used to measure protein expression levels. Briefly, polyvinylidene fluoride (PVDF) membranes were incubated with various primary antibodies at 4 °C overnight (14–16 h) and subsequently incubated with secondary antibodies. The primary and secondary antibodies used in this study were anti-MLKL (1:1000), anti-GSDMD (1:1000), anti-caspase-8 (1:1000), anti-caspase-1 (1:1000), anti-caspase-3 (1:1000), anti-BAK (1:1000), anti-FADD (1:1000), anti-caspase-9 (1:1000), anti-GSDME (1:1000), anti-IFN-α (1:1000), anti-IL-1β (1:1000), anti-RIP1 (1:1000), anti-RIP3 (1:1000), anti-TLR4 (1:1000), and GAPDH (1:1000). The secondary antibodies used were anti-mouse and anti-rabbit HRP-conjugated antibodies (ABclonal, 1:5000). After the membranes were washed thoroughly with TBST, they were incubated with secondary antibody at room temperature 25°C for 1 h. All protein bands were displayed using an enhanced chemiluminescence (ECL) assay kit with the aid of a multifunctional imager (Bio-Rad Laboratories, Hercules, CA, USA) and analyzed using ImageJ 1.8.0 (64-bit) (win-64) software.

### 2.4. Immunohistochemistry (IHC) and Immunofluorescence (IF)

The expression levels of key proteins in the liver were measured using immunohistochemistry and immunofluorescence staining. The liver tissue sections were dewaxed and hydrated, and citrate buffer was used to perform antigen retrieval: the sections were then treated with 85% methanol and 30% H_2_O_2_ to inactivate endogenous enzymes and blocked with 10% horse serum. For immunofluorescence, the sections were hydrated using xylene, after which they were placed in graded alcohol; subsequently, overnight incubation at 4 °C with the primary antibody was performed. Then, the sections were incubated with the secondary antibody at room temperature for 1 h. The results were observed with a light or fluorescence microscope and analyzed with ImageJ software. Thus, the expression levels of key proteins were determined through immunohistochemistry, and images were captured using a fluorescence microscope (Leica Microsystems, Wetzlar, Germany).

### 2.5. Histopathological Evaluation

Liver tissue sections were properly treated by immobilization, dehydration (made transparent in xylene and dehydrated with different gradients of ethanol), and paraffin embedding for sectioning (sliced into sections of 4 μm). Sections were then stained with hematoxylin and eosin (H&E). In the final step, neutral gum was used to seal and fix the tissue sections. The prepared sections were subsequently placed under a DM1000 optical microscope (Leica Microsystems, Wetzlar, Germany) for observation and imaging. The following scoring system was used to assess the severity of liver injury: Score 0: No significant abnormalities, Score 1: Minimal hepatocellular necrosis and scattered inflammatory cells, Score 2: Focal necrosis with moderate inflammatory infiltration, Score 3: Extensive necrosis with severe inflammatory infiltration and disruption of liver architecture. To minimize bias, investigators were blinded to group allocation during histopathological scoring and data analysis. Tissue sections were coded with random numbers, and the codes were revealed only after scoring was completed.

### 2.6. TUNEL Detection

TUNEL staining was carried out in accordance with the instructions provided by the manufacturer of the TUNEL staining kit, and the level of apoptosis in liver cells was assessed by calculating the ratio of TUNEL-positive cells to the total number of cells. Finally, the slices were observed and analyzed using a DM1000 optical microscope (Leica Microsystems, Wetzlar, Germany). The proportion of TUNEL-positive cells was calculated by counting the numbers of positive and negative cells. The counts of the three different fields of view were averaged to calculate the final percentage.

### 2.7. PAS Detection

PAS staining was performed according to the instructions of the manufacturer of the PAS staining kit, and polysaccharides, glycoproteins, and glycolipids were detected by observing the prepared sections using an optical microscope (DM1000, Leica Microsystems, Wetzlar, Germany).

### 2.8. Statistical Analysis

The data were obtained from at least three independent experiments, and the values represent the mean. All the statistical analyses were performed using the statistical software GraphPad Prism (version 7.00). The data are expressed as the mean ± SD (n = 8). The Shapiro–Wilk test and Levene’s test were used to assess the normality and homogeneity of variance of the data, respectively. Data with a Shapiro–Wilk test *p* > 0.05 were considered normally distributed, and a Levene’s test *p* > 0.05 indicated equal variances. Statistical differences among multiple groups were determined using one-way ANOVA followed by Tukey’s multiple comparisons test. Significant differences were denoted as # *p* < 0.05 and ## *p* < 0.01. Differences between two groups were analyzed using an unpaired *t*-test, with significance defined as * *p* < 0.05 and ** *p* < 0.01. “*” represents a statistically significant difference between the control and *Salmonella*-infected groups (* *p* < 0.05, ** *p* < 0.01). “#” indicates a statistically significant difference between the treatment and *Salmonella*-infected groups (# *p* < 0.05, ## *p* < 0.01).

## 3. Results

### 3.1. Salmonella Infection Results in Liver Damage in Mice

**Figure 1:**
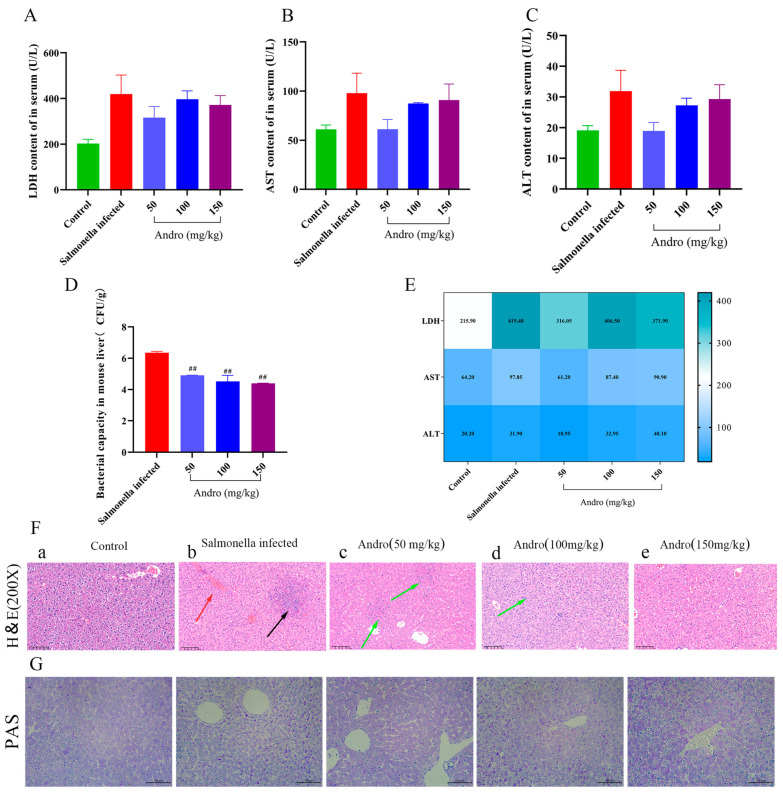
Effects on blood liver-related indices, liver bacterial load and liver tissue structure and function in mice infected with *Salmonella*. (**A**–**C**) Changes in liver-related indices in blood. (**D**) Analysis of the bacterial load in mouse livers (CFU/g). (**E**) Heatmap of the levels of enzymes related to liver injury. (**F**) Hematoxylin–eosin (H&E) staining (200×), red arrows show inflammatory cell infiltration, the black arrow shows liver cell death and aggregation, and the green arrow indicates an improvement in symptoms with medication. (**G**) PAS staining of the liver. All the data are expressed as the mean ± standard deviation (SD); “#” indicates a statistically significant difference between the *Salmonella*-infected and treatment groups (## *p* < 0.01). HE and PAS: Scale bar = 100 μm.

To explore the effect of *Salmonella* on liver damage in mice, we measured serum levels of AST, LDH and ALT, which are blood markers associated with the liver. Compared with the *Salmonella*-infected group, the levels of AST, LDH and ALT were significantly decreased in the andrographolide-treated group (Figure 1A–C). Additionally, andrographolide treatment significantly decreased the level of *Salmonella* colonization in the liver (Figure 1D).

*Salmonella* infection induced histopathological damage of the liver in mice (Figure 1F). Normal hepatocytes were structurally intact with a homogeneous interstitial space, and the hepatic sinusoids were intact with no obvious abnormalities (Figure 1F(a)). In contrast, the *Salmonella*-infected group exhibited hepatocyte death, nuclear pyknosis, marked inflammatory cell infiltration in the central vein, and hemorrhage (Figure 1F(b)). However, treatment with different concentrations of andrographolide significantly ameliorated clinical signs and reduced hepatocyte death and hemorrhage in *Salmonella*-infected mice (Figure 1F(c)). Collectively, these results indicate that andrographolide exerts hepatoprotective and antibacterial effects against *Salmonella*-induced liver injury.

Furthermore, PAS staining revealed that the number of glycogen granules (purplish color) was significantly decreased in the *Salmonella*-infected group but markedly increased in the andrographolide-treated group (Figure 1G). These results indicate that andrographolide has a symptom-reducing effect on *Salmonella*-induced liver injury.

### 3.2. Andrographolide Mitigated Salmonella-Induced Liver Apoptosis in Mice

**Figure 2:**
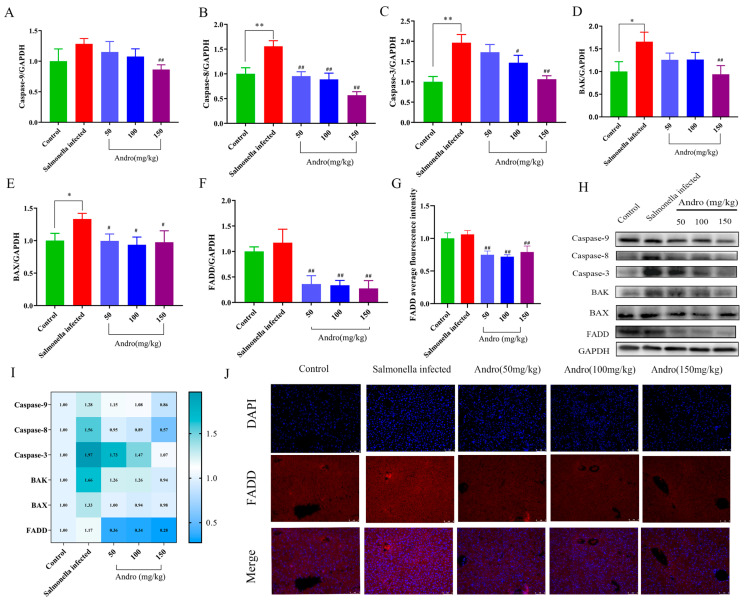
Effects of *Salmonella* infection and andrographolide treatment on liver tissue apoptosis in mice. (**A**–**F**) Protein levels of apoptosis-related genes. (**G**) Immunofluorescence analysis of FADD. (**H**) Western blot analysis of apoptosis-related proteins. (**I**) Heatmap of apoptosis-related protein levels. (**J**) Immunofluorescence of FADD. All the data are expressed as the mean ± standard deviation (SD); “*” represents a statistically significant difference between the control and *Salmonella*-infected group (* *p* < 0.05, ** *p* < 0.01). “#” indicates a statistically significant difference between the *Salmonella*-infected and treatment groups (# *p* < 0.05, ## *p* < 0.01). FADD: Scale bar = 2 mil.

To further explore the mechanism by which andrographolide alleviates liver damage induced by *Salmonella*, we measured the apoptosis-related indicators. These results showed that *Salmonella* infection significantly increased the expression levels of caspase-9, caspase-8, caspase-3, Bax, Bak and FADD proteins. However, treatment with andrographolide resulted in significant decreases in the protein expression levels of apoptosis-related markers compared with the *Salmonella*-infected group (Figure 2A–F). These findings suggest that andrographolide has a therapeutic effect on the liver and alleviates *Salmonella*-induced apoptosis. The immunofluorescence results confirmed these findings (Figure 2J).

### 3.3. Andrographolide Downregulated the Expression of Apoptosis-Related Genes in Mouse Hepatocytes Induced by Salmonella Infection

**Figure 3:**
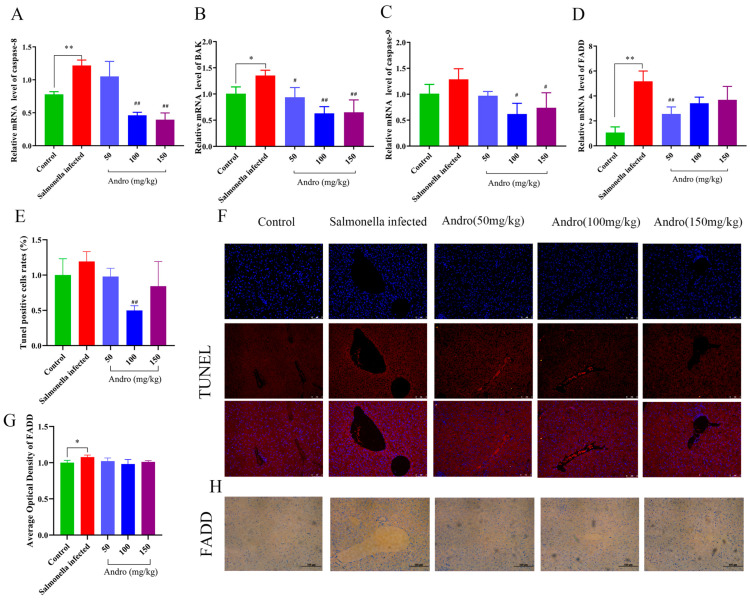
Effects of *Salmonella* infection and andrographolide treatment on liver tissue apoptosis in mice. (**A**–**D**) Relative mRNA levels of genes related to apoptosis. (**E**) Proportion of cells that were positive for TUNEL. (**F**) TUNEL staining of the liver. (**G**) Immunohistochemical analysis of FADD expression. (**H**) Immunohistochemical images of FADD expression. All the data are expressed as the mean ± standard deviation (SD); “*” represents a statistically significant difference between the control and *Salmonella*-infected groups (* *p* < 0.05, ** *p* < 0.01). “#” indicates a statistically significant difference between the *Salmonella*-infected and treatment groups (# *p* < 0.05, ## *p* < 0.01). TUNEL: Scale bar = 2 mil. FADD: Scale bar = 100 μm.

To investigate the mechanism by which andrographolide alleviates *Salmonella*-induced liver injury, we also measured other apoptosis-related indicators. These results demonstrated that, compared with the *Salmonella* infection group, treatment with different concentrations of andrographolide significantly reduced the mRNA expression levels of *caspase-9*, *caspase-8*, *BAK*, and *FADD* (Figure 3A–D). Additionally, TUNEL staining revealed increased hepatocyte cell death in the *Salmonella* infection group, which was significantly reduced in the andrographolide treatment group (Figure 3E,F). The immunohistochemical findings confirmed these observations (Figure 3G,H). These results suggest that *Salmonella* infection induced apoptosis in mouse hepatocytes, and this effect was ameliorated by andrographolide treatment.

### 3.4. Andrographolide Alleviates the Pyroptosis of Mouse Liver Cells Caused by Salmonella Infection

**Figure 4:**
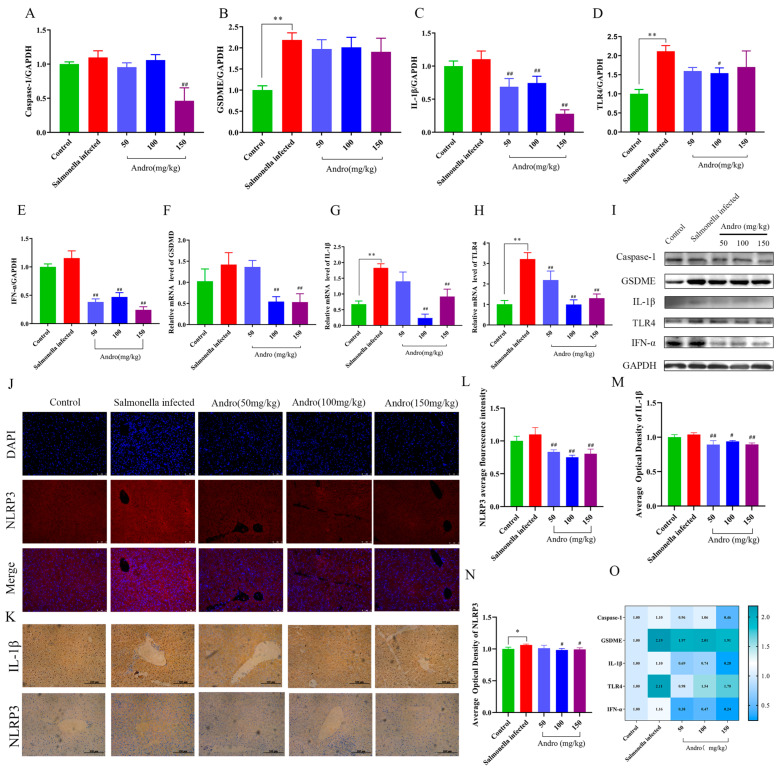
Effects of *Salmonella* infection and andrographolide treatment on liver tissue pyroptosis in mice. (**A**–**E**) Protein levels of genes associated with pyroptosis. (**F**–**H**) Relative mRNA levels of pyroptosis-related genes. (**I**) Western blot analysis of pyroptosis-related proteins. (**J**) Immunofluorescence of NLRP3. (**K**) Immunohistochemical images of IL-1β and NLRP3 expression. (**L**) Immunofluorescence analysis of NLRP3. (**M**,**N**) Immunohistochemical analysis of IL-1β and NLRP3 expression. (**O**) Heatmap of protein levels associated with pyroptosis. All the data are expressed as the mean ± standard deviation (SD); “*” represents a statistically significant difference between the control and *Salmonella*-infected groups (* *p* < 0.05, ** *p* < 0.01). “#” indicates a statistically significant difference between the *Salmonella*-infected and treatment groups (# *p* < 0.05, ## *p* < 0.01). NLRP3 immunofluorescence: Scale bar = 2 mil. IL-1β and NLRP3: Scale bar = 100 μm.

Bacterial infection leads to caspase-1-dependent pore formation and the release of proinflammatory cytokines (including IL-1β and IL-18), which increases the expression of pyroptosis-related proteins and ultimately results in cell swelling and rapid plasma membrane rupture [27]. To further explore the mechanism underlying the protective effect of andrographolide against *Salmonella*-induced liver injury in mice, we also measured indicators related to pyroptosis. These results demonstrated that *Salmonella* infection markedly upregulated the protein levels of caspase-1, GSDME, IL-1β, TLR4, and IFN-α. However, these protein levels were markedly reduced in the andrographolide-treated group (Figure 4A–E). Compared with the *Salmonella*-infected group, the andrographolide treatment group exhibited reduced mRNA expression levels of *GSDMD*, *IL-1β*, and *TLR4* in the liver of mice (Figure 4F–H). These findings are consistent with the above results. Furthermore, the immunofluorescence-positive areas for NLRP3 were reduced in the andrographolide-treated group compared with the *Salmonella*-infected group (Figure 4J). In addition, to further validate pyroptosis, the expression levels of NLRP3 and IL-1β were detected by immunohistochemistry. The results showed that the expression of these proteins was significantly decreased in the andrographolide-treated group compared with the *Salmonella*-infected group (Figure 4M,N). The heatmap showed that, at the protein level, the expression of pyroptosis-related proteins was significantly higher in the *Salmonella* infection group than in the andrographolide treatment group (Figure 4O). These findings demonstrate that *Salmonella* infection can lead to hepatocyte pyroptosis and that andrographolide treatment is effective in attenuating this process.

### 3.5. Salmonella Infection Induced Necroptosis in Mouse Hepatocytes, and Andrographolide Attenuated This Effect

**Figure 5:**
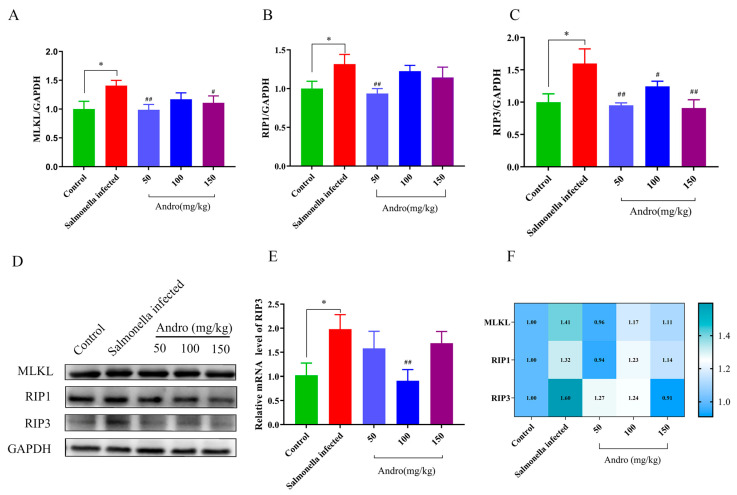
Effects of *Salmonella* infection and andrographolide treatment on necroptosis of liver tissue in mice. (**A**–**C**) Necroptosis-related protein levels. (**D**) Western blot analysis of proteins related to necroptosis. (**E**) Relative mRNA levels of genes related to necroptosis. (**F**) Heatmap of protein levels associated with necroptosis. All the data are expressed as the mean ± standard deviation (SD); “*” represents a statistically significant difference between the control and *Salmonella*-infected groups (* *p* < 0.05). “#” indicates a statistically significant difference between the *Salmonella*-infected and treatment groups (# *p* < 0.05, ## *p* < 0.01).

To explore the effects of *Salmonella* infection on necroptosis in the liver, we therefore investigated the expression levels of the necroptosis markers MLKL, RIP1, and RIP3 (Figure 5A–C). Interestingly, the expression levels of RIP1 and RIP3 were significantly upregulated in the *Salmonella*-infected group, suggesting that these proteins may be involved in the necroptosis of mouse hepatocytes. Notably, *Salmonella* infection also significantly increased the expression level of MLKL. In addition, the *RIP3* mRNA expression level was significantly decreased in the andrographolide-treated group compared with the *Salmonella*-infected group (Figure 5E). Therefore, our results demonstrated that the expression trends of necroptosis marker proteins were consistent with those of pyroptosis and apoptosis. These data suggested that *Salmonella* infection induced necroptosis of hepatocytes in mice, and andrographolide attenuated this effect.

### 3.6. The Relationships Among Pyroptosis, Apoptosis, and Necroptosis

**Figure 6:**
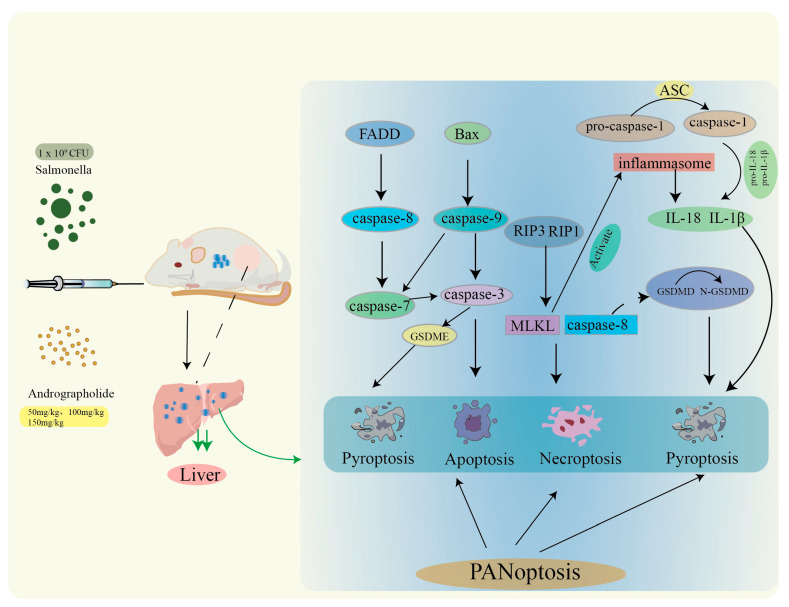
Following drug administration in mice, interactions among pyroptosis, apoptosis, and necroptosis occur in the liver. FADD and BAX mediate the intrinsic and extrinsic cell death pathways, respectively. Caspase-3, caspase-7, and caspase-9 are involved in two distinct pathways: one leads to apoptosis, whereas the other leads to pyroptosis via GSDME. RIP3, RIP1 and MLKL mediate necroptosis. MLKL activates inflammasomes, thereby activating caspase-1, IL-18 and IL-1β, which induce pyroptosis.

Apoptosis, pyroptosis, and necroptosis are three distinct forms of regulated cell death that differ in their morphological features, molecular mechanisms, and inflammatory outcomes. However, these three pathways are not mutually exclusive, they converge through the PANoptosome to form PANoptosis, a key host defense mechanism implicated in *Salmonella* infection (Figure 6). The present study demonstrated that andrographolide regulated the markers of apoptosis, pyroptosis, and necroptosis in hepatocytes of infected mice, and these three pathways are associated with PANoptosis.

## 4. Discussion

*Salmonella* bacteria are major enteric pathogens that infect humans and animals, and *Salmonella* typhi is among the most common isolates in clinical practice. Previous studies have shown that liver injury is usually accompanied by intestinal barrier dysfunction and that these two organs interact through the gut–liver axis. Furthermore, intestinal epithelial damage may represent the main pathological mechanism underlying the enterohepatic dissemination of *Salmonella* [28,29]. *Salmonella* is an enteric pathogen that colonizes the intestine. We therefore explored whether, after accumulating in large numbers in the intestine, *Salmonella* can induce liver infection via the hepatic portal vein. The above research findings confirmed this finding. Andrographolide has antibacterial, hepatoprotective and anticancer properties. The compound may inhibit cancer cell development by inducing autophagy [30]. It has also been shown that andrographolide can inhibit cancer cell proliferation and metastasis by enhancing mitochondrial function, thereby promoting ferroptosis induction. Thus, andrographolide is a promising drug for tumor treatment [31]. Notably, few studies have investigated the role of andrographolide in PANoptosis. Here, our findings demonstrate that andrographolide alleviates liver injury in *Salmonella*-infected mice by downregulating the expression of markers associated with hepatocellular apoptosis, pyroptosis, and necroptosis.

*Salmonella* is an intestinal colonizing bacterium, but it can also cause damage to other organs. Studies have shown that *Salmonella typhimurium* can reside in Kupffer cells in the liver, which regulate and attenuate antimicrobial inflammation by triggering T cells [32,33,34]. A recent study revealed that *Salmonella typhimurium* can adapt to its environment through different mutations specific to the liver and spleen during persistent infection in mice infected with drug-resistant strains [35]. Furthermore, a study revealed that reducing inflammation through *Salmonella* plasmid virulence c (*Spvc*) can cause systemic infections [36]. In addition, Song et al. [8] reported that the liver is susceptible to gut microbes and metabolites and that *Salmonella* infection usually leads to an acute inflammatory response in the liver, hepatomegaly enlargement and congestion. It has been reported that *Salmonella typhimurium* can compromise the intestinal barrier and increase intestinal permeability, thereby promoting bacterial or toxin translocation across it and subsequently inducing liver injury [37]. Our research is consistent with these findings. These results further support our findings that *Salmonella typhimurium* caused liver damage in mice. Additionally, tissue bacterial load directly reflects the degree of bacterial infection in tissues and is therefore among the most important indicators for assessing *Salmonella*-induced liver damage. In this study, we report that *Salmonella typhimurium* infection results in weight loss and decreased food intake. To assess *Salmonella*-induced liver injury in mice, we measured markers of liver damage, and the results confirmed this effect (Figure 1A–C). In addition, we treated the liver injury with andrographolide, and the results suggested that andrographolide may alleviate *Salmonella* infection through the PANoptosis pathway. Due to damage to the intestinal barrier, *Salmonella typhimurium* may disseminate through the bloodstream to the liver and spleen, leading to hepatosplenomegaly associated with apoptosis and inflammatory infiltration. Our TUNEL staining results indicate that *Salmonella* infection induces high rates of apoptosis in liver cells. In addition, compared with the *Salmonella* infection group, we observed that infection was significantly alleviated in the andrographolide treatment group. This was evidenced by decreased hepatocyte apoptosis and inflammatory cell infiltration. Histologically, this effect was confirmed by H&E staining (Figure 1F). Destruction of liver tissue leads to hepatocellular damage and increased permeability of the plasma membrane in hepatocytes, which is manifested by the release of enzymes (ALT, AST and LDH) from hepatocytes into the bloodstream. Our results revealed that the levels of the enzymes ALT, AST and LDH were increased in mice of the *Salmonella*-infected group compared with those in the treated group. These findings are consistent with the results reported by Wang et al. [38] regarding the effect of cinnamaldehyde against *Salmonella typhimurium* in mouse hepatocytes. *Salmonella typhimurium*-induced liver injury manifested as an elevated liver index in mice. This is because microorganisms and their metabolites may accumulate in the liver, acting as mediators via the gut–liver axis and bile ducts, thereby leading to liver injury and inflammation. Notably, *Salmonella* can induce liver injury in mice through its anti-inflammatory and antioxidant effects [8]. Consistent with our study, these findings appear to support a role for *Salmonella* exposure in liver injury in mice.

Bacterial infections often cause inflammation. There are two types of pyroptosis: moderate pyroptosis helps the immune system clear pathogens and damaged cells, whereas excessive pyroptosis may aggravate the inflammatory response and cause damage to normal cells and tissues [39]. Pyroptosis is involved in many diseases, including liver disease. Previous studies have shown that NLRs and AIM2 interact with ASC to recruit pro-caspase-1, forming an inflammasome, which then cleaves pro-caspase-1 to produce caspase-1. Caspase-1 subsequently induces the degradation of the protein gasdermin D (GSDMD) and the release of its N-terminal fragment GSDMD-N [40], which leads to the formation of cell membrane pores, causing cell contents to leak out. In addition, caspase-1 promotes the maturation of interleukin-18 (IL-18) and IL-1β as well as the release of these proinflammatory factors, thereby further mediating inflammatory signaling pathways [41]. *Salmonella* SpvC mediates the inhibition of pyroptosis in *Salmonella*-infected macrophages through its threonine lyase activity, which significantly reduces the levels of the pyroptosis-related protein markers NLRP3 and GSDMD [36]. Based on these findings, we examined pyroptosis in hepatocytes and demonstrated that andrographolide may ameliorate liver injury induced by *Salmonella* infection by modulating the expression of markers associated with pyroptosis. The expression levels of the markers caspase-1, GSDMD, GSDME, and IL-1β, which are associated with pyroptosis, were analyzed by Western blotting and RT-qPCR. The results revealed elevated expression levels of these pyroptosis proteins in the *Salmonella*-infected group. In addition, we investigated hepatocyte apoptosis in infected mice. In our experiments, we found that caspase-3 activity was significantly increased during *Salmonella typhimurium* infection, and that treatment with different concentrations of andrographolide significantly decreased caspase-3 activity. These findings are consistent with those of Yin et al. [42], who reported that cinnamaldehyde exerted antioxidant and antiapoptotic effects in the hepatocytes of young chickens infected with *Enterococcus faecalis*. Thus, we found that andrographolide treatment may exert an anti-apoptotic effect on mouse hepatocytes attacked by *Salmonella*, as evidenced by a significant reduction in the protein expression levels of relevant apoptotic markers.

Furthermore, our results revealed that after infecting mice with *Salmonella typhimurium*, the massive accumulation of RIP3 might upregulate the phosphorylation of MLKL, which in turn could induce necroptosis in hepatocytes. This is consistent with the results reported by Dong et al. [43], who demonstrated that *Salmonella typhimurium* infection promotes necroptosis via the *spvB* gene. Thus, we propose that *Salmonella* infection may also induce necroptosis in hepatocytes.

According to relevant studies, andrographolide exerts a good therapeutic effect on inflammatory diseases [44], including liver disease, joint disease, respiratory disease, inflammatory bowel disease, and inflammatory skin disease. Therefore, andrographolide appears to play an essential role in the treatment of *Salmonella* infection in the context of resistance. In addition to these diseases, it also plays important roles in the response to malignant tumors in different organs, such as liver cancer, stomach cancer, and rectal cancer. Andrographolide has been extensively studied in cancer, inflammation, and other diseases. However, few studies have focused on liver diseases induced by *Salmonella* infection. Through these experiments, andrographolide appears to confer protection against liver injury. As for the underlying cause, we propose that the drug may exert its therapeutic effects through the modulation of markers associated with apoptosis, pyroptosis, and necroptosis. Apoptosis-, pyroptosis-, and necroptosis-related molecules were significantly increased in the *Salmonella*-infected group compared with the control group, but significantly decreased after andrographolide treatment compared with the *Salmonella*-infected group, indicating a potential inhibitory effect. In addition, Asiatic acid and andrographolide have been reported to attenuate hippocampal damage by inhibiting neuroinflammation induced by *Salmonella typhimurium* infection, while also reducing the number of invading bacteria [21]. These findings corroborated our observations, suggesting that andrographolide can exert its protective effects against *Salmonella*-induced liver injury and inflammation in mice by modulating markers associated with apoptosis, pyroptosis, and necroptosis. We found that andrographolide treatment significantly reduced the expression levels of IL-1β, and IFN-α, suggesting an anti-inflammatory effect. Andrographolide is a diterpenoid lactone isolated from *Andrographis paniculata* that has been shown to possess anti-inflammatory activity in neurodegenerative diseases [45]. In addition, studies have confirmed that andrographolide and Ajwain are potential alternative antibiotics for combating *Salmonella* gallinarum in poultry environments [22]. In our work, *Salmonella* was selected to construct a mouse model. B-cell lymphoma-2 (Bcl-2) and Bcl-2-associated X protein (Bax) promote stressor-induced apoptosis, and Bax can cause programmed cell death by permeabilizing the outer mitochondrial membrane and subsequently initiating a caspase cascade [46]. In *Salmonella*-infected mice, liver injury was induced, and we suggest that andrographolide appears to downregulate the expression levels of proteins associated with markers of apoptosis (caspase-3/9, Bax, Bak, caspase-8, and FADD). These results are similar to those reported by Luo et al. [47], who found that andrographolide significantly attenuated Mycoplasma gallisepticum (MG)-induced inflammation and apoptosis in chickens by downregulating the expression of Bax and caspase-3/9. According to the above results, andrographolide plays an important role in regulating apoptosis. This study confirms that the application of andrographolide at different concentrations can effectively improve clinical symptoms. This phenomenon may be attributed to the ability of andrographolide to inhibit *Salmonella typhimurium*-induced PANoptosis, which may contribute to its protective effects against liver injury.

Our study has several limitations. *Salmonella typhimurium* is found mainly in the gastrointestinal tract. However, few studies have investigated how it causes liver injury. The process or mechanism by which *Salmonella* causes liver damage and inflammation needs to be further explored so that effective measures can be implemented to treat the disease. Furthermore, there are relatively few studies investigating the alleviation of *Salmonella* infection in mice by andrographolide through inhibition of the PANoptosis pathway. In the future, we will extend our research to an in-depth investigation of the PANoptosome, a key complex associated with PANoptosis, as well as other drugs as alternatives to antibiotics for *Salmonella* infection.

## 5. Conclusions

In summary, our findings provide evidence for a potential role of andrographolide in attenuating *Salmonella*-induced liver damage in mice, possibly via the inhibition of PANoptosis. In addition, andrographolide modulates markers of apoptosis, pyroptosis, and necroptosis, suggesting potential regulation of PANoptosis-related pathways. Our findings emphasize the potential role of different concentrations of andrographolide in the treatment of liver injury and elucidate its associated pathways, which may provide important ideas and clues for antibiotic substitutes.

## Data Availability

The original contributions presented in this study are included in the article/Supplementary Material. Further inquiries can be directed to the corresponding author.

## Supplementary Materials

The following supporting information can be downloaded at https://www.mdpi.com/article/10.3390/microorganisms14040936/s1.
