# Supplementary material for: Andrographolide Alleviates Liver Damage Caused by Salmonella in Mice by Inhibiting the PANoptosis Pathway
Source: Microorganisms. 2026 Apr 21;14(4):936. doi: 10.3390/microorganisms14040936 (PMC13118790; doi:10.3390/microorganisms14040936)
Supplement: Supplementary file 1 [file microorganisms-14-00936-s001.zip › microorganisms-4217547-supplementary.pdf]

Table S1.

Gene	Forward primer (5′-3′)	Reverse primer (5′-3′)
GSDMD	aaatcctgcaacagcttcgg	tcaccatcttcttccggctt
Caspase-8	cgtctatggaacggatggga	gccattcccagcagaaagtc
TLR4	aggttgagaagtccctgctg	gtttgctcaggattcgaggc
IL-1β	tcaggcaggcagtatcactc	agctcatatgggtccgacag
BAK	caacagcatcttgggtcagg	gtagacgtacagggccagac
Caspase-9	cacggctttgatggagatgg	gccatggtctttctgctcac
RIP3	cgggaaacagtgtgtgacag	ctcggagacagcagcatcta
FADD	gcaacgatctgatggagctc	gctgcagtagatcgtgtcg
